# Relation between DNA ionization potentials, single base substitutions and pathogenic variants

**DOI:** 10.1186/s12864-019-5867-y

**Published:** 2019-07-16

**Authors:** Fabrizio Pucci, Marianne Rooman

**Affiliations:** 1 Computational Biology and Bioinformatics, Université Libre de Bruxelles, Roosevelt Ave. 50, Bruxelles, 1050 Belgium; 2 John von Neumann Institute for Computing, Jülich Supercomputer Centre, Forschungszentrum Jülich, Jülich, 52428 Germany

**Keywords:** Single base substitutions, Pathogenic mutations, Charge transport, DNA base stacking, Vertical ionization potential

## Abstract

**Background:**

It is nowadays clear that single base substitutions that occur in the human genome, of which some lead to pathogenic conditions, are non-random and influenced by their flanking nucleobase sequences. However, despite recent progress, the understanding of these "non-local" effects is still far from being achieved.

**Results:**

To advance this problem, we analyzed the relationship between the base mutability in specific gene regions and the electron hole transport along the DNA base stacks, as it is one of the mechanisms that have been suggested to contribute to these effects. More precisely, we studied the connection between the normalized frequency of single base substitutions and the vertical ionization potential of the base and its flanking sequence, estimated using MP2/6-31G* *ab initio* quantum chemistry calculations. We found a statistically significant overall anticorrelation between these two quantities: the lower the vIP value, the more probable the substitution. Moreover, the slope of the regression lines varies. It is larger for introns than for exons and untranslated regions, and for synonymous than for missense substitutions. Interestingly, the correlation appears to be more pronounced when considering the flanking sequence of the substituted base in the 3’ rather than in the 5’ direction, which corresponds to the preferred direction of charge migration. A weaker but still statistically significant correlation is found between the ionization potentials and the pathogenicity of the base substitutions. Moreover, pathogenicity is also preferentially associated with larger changes in ionization potentials upon base substitution.

**Conclusions:**

With this analysis we gained new insights into the complex biophysical mechanisms that are at the basis of mutagenesis and pathogenicity, and supported the role of electron-hole transport in these matters.

**Electronic supplementary material:**

The online version of this article (10.1186/s12864-019-5867-y) contains supplementary material, which is available to authorized users.

## Background

The understanding of the biophysical mechanisms that drive single DNA base substitutions in different regions of the genome is one of the key questions of the post-genomic era. Indeed, the effects of these mutation processes are potentially responsible for a range of diseases such as cancer and various neurodevelopment disorders. The rationalization of these mechanisms is very complex, since single base substitutions (SBSs) can be triggered by a wide range of factors, *e.g.* chemical species, physical agents or enzymes, through mechanisms such as base deamination, base depurination, or tautomeric shifts. These effects moreover depend on the nucleotide sequence context [1, 2].

The analysis of the large quantity of available genomic data has allowed to firmly state that SBSs do not occur randomly along the genome. For example, one of the well-known mutational signatures observed in cancer genomes occurs at mCpG dinucleotides [3, 4]. Methylated cytosines (mC) undergo more frequently spontaneous deamination than unmethylated cytosines, which leads to the C →T transition with higher probability. The second signature, which has been observed in different cancer types, occurs at TpC dinucleotides in which the cytosine is mutated through transition or transversion. It has been related to the overactivity of the APOBEC cytidine deaminases [5, 6]. Other signatures are specific to certain types of cancers or diseases [7–9]. For example, the C:G →A:T transversion is a well-known mutation in lung cancer induced by tobacco carcinogens. The "UV signature", namely C →T mutations at dipyrimidine sites, is caused by ultraviolet light via cyclobutane pyrimidine dimer formation and is commonly found in melanomas.

It became recently clear that there is an important effect of the flanking base sequence on SBSs [2], which appears to extend beyond the dinucleotide units [10]. However, the mechanisms triggering these "non-local" effects are far from clear. Their comprehension is of primary importance and would lead to deep insights into mutagenesis phenomena and the deleteriousness of genetic variations.

In this context, the physical phenomena of electron-hole transfer along the DNA stack comes into play. Exposure to high-energy radiations or to reactive oxygen species generated as by-products of the cellular metabolism can lead to DNA ionization through the creation of electron holes. These holes then migrate along the DNA stack until they remain (more or less) localized in a potential well [11–13].

Although some electron holes have solely a damaging effect and need to be repaired by specific enzymes [14], others seem to have a positive role as suggested more than a decade ago by the finding that the amount of reactive oxygen species likely to create such holes is regulated by specific proteins and is, for example, higher during cell differentiation [15]. The important role played by electron-hole transfer in various biological processes appears increasingly clear [16, 17] and more specifically, in the sequence dependence of the SBSs [2, 12, 13, 18] and their possible pathogenicity [12, 19].

Important steps towards the understanding of long-distance hole transfer in DNA have been achieved. However, large parts of the picture are still lacking. Indeed, experimental measures are challenging due to the complexity of working at the nanoscale [20, 21], and accurate quantum chemistry calculations are extremely heavy for these systems [22, 23].

In this paper, we further analyzed the relationship between the electron-hole transfer along the DNA stack, the sequence-dependence of the mutability and the pathogenicity of the mutations. For that purpose, we calculated the vertical ionization potential (vIP) of all short nucleobase sequences and correlate these to the presence of benign and/or deleterious mutations occurring in cancer and inherited disorders, with the goal of confirming the prominent contribution of hole migration in these matters.

## Results

### Mutation frequencies in the SBS dataset

We have set up a dataset of single base substitutions which occur in genes, *i.e.* in exons, introns or untranslated regions (UTRs), and are annotated as pathogenic, benign, of unclear significance, etc, as described in Methods. In exons, we distinguish between synonymous and missense variants.

Let us start by analyzing which of the base pairs and which of the dinucleotide or trinucleotide stacks (also called doublets and triplets) mutate significantly more or less frequently than expected from random substitutions in the different gene regions. Note that the triplets that we consider are defined as three successive bases and usually do not correspond to codons, even if located in exons. The results are summarized in Table 1, and reported exhaustively in Additional file 2: Table S4.

**Table 1:** Normalized SBS frequencies, defined as the ratio between the frequencies of mutations in the nucleobase motifs and the observation frequencies of the same motifs in exons (with a distinction between missense and synonymous SBSs), introns and UTRs

Missense		Synonymous		Intron		UTR	
G:C	1.27	G:C	1.39	G:C	1.53	G:C	1.31
**C**pG	3.33	**C**pG	5.40	**C**pG	8.70	**C**pG	4.10
**G**pG	1.74	**G**pG	2.75	**G**pG	1.97	**G**pG	1.69
C**G**T	4.87	A**C**G	6.62	C**G**C	12.80	C**G**T	5.58
C**G**G	4.35	C**C**G	6.54	C**G**G	12.48	A**C**G	4.81
C**C**G	4.21	C**G**G	6.20	C**C**G	11.70	C**C**G	4.67
A:T	0.70	A:T	0.57	A:T	0.64	A:T	0.71
**A**pG	0.54	**T**pC	0.40	**A**pA	0.53	**T**pG	0.59
**A**pA	0.50	**A**pC	0.39	**T**pA	0.51	**T**pC	0.49
C**A**A	0.44	G**A**T	0.24	C**T**A	0.40	C**A**A	0.43
T**T**G	0.33	G**A**C	0.19	C**A**A	0.37	T**T**C	0.39
G**A**A	0.30	G**A**A	0.15	G**A**A	0.32	G**A**A	0.33
R _All_	2.0	R _All_	4.7	R _All_	2.0	R _All_	2.0
R _CpG_	4.9	R _CpG_	9.4	R _CpG_	4.5	R _CpG_	3.3

The nucleotide at which the substitution occurs is in bold. The mutations that are more frequent than expected are shown in the upper section of the Table, those that are less frequent in the middle section, and the ratio of transitions to transversions (noted R) in the lower section. Exhaustive results are given in Additional file 2: Table S4

First, the well-known bias towards mutations that occur at C:G base pairs rather than at A:T [18] is clearly observed for missense, synonymous, UTR and intron mutations. Indeed, the frequency of mutations of C or G is 1.3 to 1.5 higher than expected on the basis of the frequency of C:G pairs in the corresponding region, whereas SBSs at A or T are 0.6 to 0.7 times less frequent than expected. Note that C:G pairs are slightly underrepresented in the human genome (around 40%) but not in the human exome (around 50–55%).

The preference for SBSs to occur on the C base of CpG dinucleotides is even more pronounced, with a rate that is 3 to 9 times higher than expected from the CpG frequency. CpGs are underrepresented in the whole genome, which can be attributed to the fact that they are the preferential target of DNA-methyltransferase: this enzyme methylates the cytosine that can more easily undergo the base substitution mCpG →TpG [18, 24].

The other doublet that is preferentially targeted by mutations is **G**pG (with the substituted base in bold). Its mutation rate is from 2 to 3 times higher than expected. In contrast, the least mutated dinucleotides are **T**pN and **A**pN where N=A, C or G, with a rate that is 6 to 18 times lower than the **C**pG mutation rate.

Among triplets, SBSs occur preferentially at the middle base of C**G**G, C**C**G, C**G**T, C**G**C and A**C**G, in all regions (some are just below the threshold, see Additional file 2: Table S4). At the other extreme, G**A**A and some other triplets with A- or T-base substitutions occur at rates that are up to 40 times lower than the most frequent SBS triplets.

Interestingly, the observed trends are almost identical for missense and UTR mutations, and are amplified for synonymous and intronic mutations: the most frequently mutated motifs are even more frequent, and the least frequent SBSs are even less frequent. This amplification is quite strong: for **C**pG doublets, for example, the frequency of SBSs is 9 times higher in introns than expected from the frequency of this doublet, 5 times higher for synonymous SBSs, and only 3–4 times for UTR and missense SBSs. For G**A**A triplets, which are less frequent than expected, the frequency ratio is 0.3 for missense, UTR and intron mutations, and only 0.15 for synonymous mutations. The reason of these differences is currently unclear, as mentioned in the “Discussion” section.

Finally, we observed that the majority of SBSs are transitions. Indeed, the ratio of transitions to transversions is equal to 2 in the missense, intron and UTR subgroups. These values are close to those already observed in other analyses [25, 26]. However this ratio increases up to 5 for the synonymous mutations. Moreover, it substantially increases at **C**pG sites where it reaches values between 3 and 9.

### Relation between the vIP of nucleobase motifs and the SBS frequencies

An important biophysical mechanism that can be related to the sequence dependence of the SBSs is the presence and migration of radical cations (electron holes) along the DNA molecules. Indeed, due to oxidative stress caused by physical or chemical agents, an electron can be extracted from the DNA. The electron hole then starts migrating along the aromatic rings of the stacked nucleobases, until it remains trapped in a minimum of the ionization potential. The hole can be filled by specific DNA repair proteins or, if not, trigger a base substitution.

In order to quantify these long-range effects and their relation with SBSs, we started by calculating the vIP of all 4 nucleobases, 16 nucleobase doublets, 64 triplets and 256 quadruplets using second-order Møller-Plesset perturbation theory as described in Methods. These vIP values, which are directly related to the probability of extraction of an electron from the DNA base-stacking structure, are given in Additional file 2: Table S4. We also computed the vIP of all nucleobase quintuplets by taking the mean of the vIPs of the two overlapping quadruplets, and of the sextuplets as the mean of the three overlapping quadruplets. For the single-base vIPs, we used the experimental values given in Table 2, which are slightly different from the calculated ones and yield better results in the present context, as expected; note that no experimental values are available for polynucleotide stacks.

**Table 2:** Experimental vIP values of nucleotide bases (eV) [48, 49]

Gua	Ade	Cyt	Thy
8.20 (±0.1)	8.42 (±0.1)	8.85 (±0.1)	9.10 (±0.1)

**Fig. 1:**
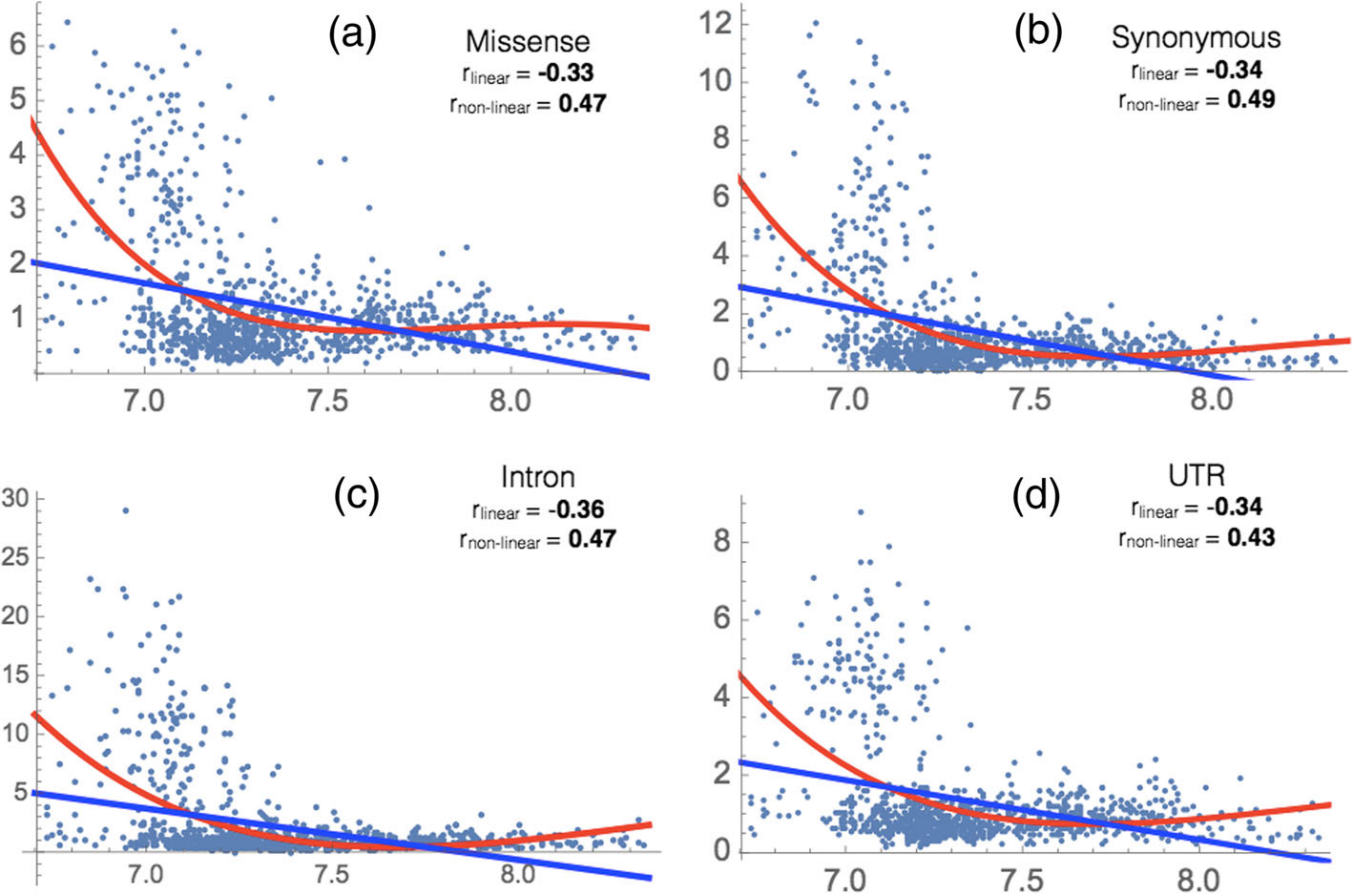
Normalized frequency of observation of SBSs as a function of the vIP (in eV) of nucleobase quintuplets NNXNN, where X indicates the SBS and N any base, for missense, synonymous, intron and UTR mutations. Both the linear (blue) and polynomial (red) regression lines are drawn. The linear (*r*_linear_) and non-linear (*r*_non-linear_) correlation coefficients between the SBS frequencies and the vIP of the quintuplets are indicated

We computed the linear and non-linear correlation coefficients between the vIP of the wild type nucleotide motifs and the frequency of observation of the motifs in the SBS dataset normalized by the frequency of their occurrences in the corresponding gene regions (see Methods). These correlations were calculated separately for synonymous and missense mutations in exons, for mutations in introns and UTRs, and for different nucleobase motif lengths, *i.e.* doublets (XN), triplets (NXN), quadruplets (NXNN), quintuplets (NNXNN) and sextuplets (NNXNNN), where X indicates the position of the nucleobase substitution in the sequence motif, and N any of the four bases. The results together with statistical significance tests are shown in Table 3, Additional file 1: Table S1-S3, Additional file 2: Table S4, Fig. 1 and Additional file 1: Figure S1-S4.

**Table 3:** Pearson’s linear correlation coefficient between the vIP of the wild-type nucleotide motifs and their normalized SBS frequency in the different gene regions

X	Doublets _XN_	Triplets _NXN_	Quadruplets _NXNN_	Quintuplets _NNXNN_	Sextuplets _NNXNNN_
**Missense mutations in exons**					
All	-0.39	-0.39 $\phantom {\dot {i}\!}{~}_{(<0.05)}$	-0.35_(<0.01)_	-0.34 $\phantom {\dot {i}\!}{~}_{(<10^{-13})}$	-0.31 $\phantom {\dot {i}\!}{~}_{(<10^{-51})}$
G	-0.23	-0.54 $\phantom {\dot {i}\!}{~}_{(<0.05)}$	-0.36 $\phantom {\dot {i}\!}{~}_{(<0.01)}$	-0.32 $\phantom {\dot {i}\!}{~}_{(<10^{-6})}$	-0.32 $\phantom {\dot {i}\!}{~}_{(<10^{-13})}$
C	-0.81	-0.71_(<0.005)_	-0.59 $\phantom {\dot {i}\!}{~}_{(<10^{-5})}$	-0.54 $\phantom {\dot {i}\!}{~}_{(<10^{-14})}$	-0.48 $\phantom {\dot {i}\!}{~}_{(<10^{-56})}$
A	0.77	0.55 $\phantom {\dot {i}\!}{~}_{(<0.05)}$	0.39 $\phantom {\dot {i}\!}{~}_{(<0.001)}$	0.36 $\phantom {\dot {i}\!}{~}_{(<10^{-6})}$	0.23 $\phantom {\dot {i}\!}{~}_{(<10^{-17})}$
T	-0.06	0.18	-0.05	-0.03	-0.07
**Synonymous mutations in exons**					
All	-0.48	-0.42 $\phantom {\dot {i}\!}{~}_{(<0.005)}$	-0.39 $\phantom {\dot {i}\!}{~}_{(<10^{-8})}$	-0.38 $\phantom {\dot {i}\!}{~}_{(<10^{-29})}$	-0.34 $\phantom {\dot {i}\!}{~}_{(<10^{-109})}$
G	-0.65	-0.67_(<0.005)_	-0.50_(<0.00005)_	-0.42 $\phantom {\dot {i}\!}{~}_{(<10^{-14})}$	-0.33 $\phantom {\dot {i}\!}{~}_{(<10^{-32})}$
C	-0.80	-0.68_(<0.05)_	-0.54 $\phantom {\dot {i}\!}{~}_{(<0.005)}$	-0.53 $\phantom {\dot {i}\!}{~}_{(<10^{-13})}$	-0.50 $\phantom {\dot {i}\!}{~}_{(<10^{-50})}$
A	-0.59	0.02	-0.09	0.00	-0.05
T	-0.87	0.00	-0.21	-0.17_(<0.05)_	-0.13_(<0.0005)_
**Mutations in introns**					
All	-0.39	-0.42_(<0.005)_	-0.38 $\phantom {\dot {i}\!}{~}_{(<10^{-5})}$	-0.36 $\phantom {\dot {i}\!}{~}_{(<10^{-24})}$	-0.33 $\phantom {\dot {i}\!}{~}_{(<10^{-78})}$
G	-0.27	-0.51_(<0.05)_	-0.36 $\phantom {\dot {i}\!}{~}_{(<0.05)}$	-0.31 $\phantom {\dot {i}\!}{~}_{(<10^{-6})}$	-0.23 $\phantom {\dot {i}\!}{~}_{(<10^{-11})}$
C	-0.78	-0.70_(<0.05)_	-0.61 $\phantom {\dot {i}\!}{~}_{(<10^{-7})}$	-0.55 $\phantom {\dot {i}\!}{~}_{(<10^{-20})}$	-0.54 $\phantom {\dot {i}\!}{~}_{(<10^{-83})}$
A	0.80	0.67_(<0.05)_	0.45_(<0.01)_	0.22_(<0.005)_	0.09 $\phantom {\dot {i}\!}{~}_{(<10^{-8})}$
T	0.07	0.41	0.34 $\phantom {\dot {i}\!}{~}_{(<0.005)}$	-0.09	-0.11
**Mutations in UTRs**					
All	-0.43	-0.39_(<0.05)_	-0.35_(<0.005)_	-0.34 $\phantom {\dot {i}\!}{~}_{(<10^{-16})}$	-0.32 $\phantom {\dot {i}\!}{~}_{(<10^{-57})}$
G	-0.18	-0.47	-0.31 $\phantom {\dot {i}\!}{~}_{(<0.05)}$	-0.25_(<0.01)_	-0.16_(<0.0001)_
C	-0.79	-0.71_(<0.05)_	-0.60_(<0.0001)_	-0.57 $\phantom {\dot {i}\!}{~}_{(<10^{-20})}$	-0.53 $\phantom {\dot {i}\!}{~}_{(<10^{-67})}$
A	0.60	0.64_(<0.01)_	0.54 $\phantom {\dot {i}\!}{~}_{(<0.05)}$	0.49 $\phantom {\dot {i}\!}{~}_{(<10^{-11})}$	0.28 $\phantom {\dot {i}\!}{~}_{(<10^{-15})}$
T	-0.13	0.33	-0.02	0.00	-0.04

X indicates the position of the mutated nucleobase and N any base. The correlation coefficients that are statistically significant and for which the null hypothesis is rejected are underlined, with the *P*-values below *α*=0.05 reported in parentheses. Note that the increase in sample size, from doublet to sextuplet motifs, also contributes to the increase of the statistical significance of the correlation

Let us first focus on the general behavior displayed by SBSs. When all possible substitutions are considered together, there is a clear, statistically significant, effect of the frequency modulation of the SBSs by the vIP of the wild-type nucleobase and its neighboring sequence. This "non-local" effect is already visible at the dinucleotide level, and clearly extends to longer sequence motifs. For doublets, the linear correlation coefficient between normalized SBS frequencies and vIP values is between −0.4 and −0.5. This correlation smoothly decreases when taking more flanking residues into account, to −0.3 for sextuplets, while becoming even more statistically significant. In other words, the lower the vIP of the sequence including the SBS, the higher the probability of the nucleotide substitution. Different biophysical mechanisms are likely to be responsible for this behavior, which will be discussed in the next section.

Interestingly, if we focus on the linear correlation between vIP and normalized SBS frequency separately for each of the four wild-type nucleobases, we see that the global anticorrelation is mainly due to the sequence motifs with a substituted guanine or cytosine. Motifs with cytosine substitutions, in particular, show the strongest anticorrelation with the vIP values, which even exceeds the correlation of guanine mutations, even though the latter base has the lowest vIP. Indeed, SBSs involving cytosine have linear correlations between -0.7 for triplets and -0.5 for sextuplets.

In contrast, motifs with a substituted adenine or thymine behave differently. They display no or even a positive linear correlation with their vIPs. More precisely, Ade-SBSs show a positive correlation, while Thy-SBSs have basically no correlation. This is true in all gene regions with a single exception, synonymous mutations in exons. The latter have a distinct behavior: substitutions involving thymine show a slight negative correlation with vIPs, while SBSs involving adenine substitutions do not correlate at all.

Let us have a closer look at Fig. 1 and Additional file 1: Figure S1-S4. Even though the linear correlation coefficients are statistically significant, it seems that the actual correlation is rather non-linear. This is confirmed by the higher values of the non-linear correlation coefficients obtained by fitting the data with a third-degree polynomial function (see Methods), which are reported in Additional file 1: Table S1. For low vIP values, the regression curve is strongly decreasing, while it is constant or slightly increases for large vIP values.

While the correlation coefficients between the vIP of the motifs and the normalized SBS frequencies show similar trends across the different gene regions, the regression slopes vary substantially. They are the lowest for missense exon mutations and UTR variants, about two times higher for synonymous exon mutations and about three times higher for mutations in introns, as seen in Table 4. This result is related to the observation that the normalized SBS frequencies have a much larger variance for synonymous and intron mutations than for missense and UTR substitutions (see Table 1). It means that, whereas the chance of having an SBS is larger for low-vIP motifs in all gene regions, this tendency is stronger for synonymous mutations and even more in introns. This leads us to conclude that different mutation mechanisms are probably involved in the different regions. We will come back to this point in the “Discussion” section.

**Table 4:** Slope of the regression lines of the normalized SBS frequency as a function of the vIP of the corresponding wild-type nucleotide motifs in the different gene regions

X	Doublets _XN_	Triplets _NXN_	Quadruplets _NXNN_	Quintuplets _NNXNN_	Sextuplets _NNXNNN_
**Missense mutations in exons**					
All	-0.6	-1.1	-1.1	-1.3	-1.5
**Synonymous mutations in exons**					
All	-1.4	-1.8	-2.1	-2.4	-2.6
**Mutations in introns**					
All	-1.8	-3.7	-3.7	-4.4	-5.3
**Mutations in UTRs**					
All	-0.9	-1.4	-1.4	-1.5	-1.7

X indicates the position of the mutated nucleobase and N any base

In summary, the correlation between normalized SBS frequencies and vIP values is highly statistically significant and extends along the nucleobase stack up to sextuplets at least. This strongly supports the importance of the electronic properties of the DNA and the charge migration along the DNA stack in the modulation of the SBSs.

### 5’-3’ asymmetry of the flanking sequence in the vIP-SBS frequency correlations

We analyzed whether the base sequence flanking an SBS in the 5’ direction is more, or less, informative than the sequence in the 3’ direction. For that purpose, we computed the SBS frequency of the nucleotide sequences 5’-XN-3’, 5’-XNN-3’ and 5’-XNNN-3’ and of the "inverse" sequences 5’-NX-3’, 5’-NNX-3’, 5’-NNNX-3’. We then computed the difference of the correlation coefficients *r*_XN..N_ and *r*_N..NX_ between vIPs and normalized SBS frequencies. The results are given in Table 5.

**Table 5:** 5’-3’ directional asymmetry of the SBS flanking sequences, observed from differences in Pearson’s linear correlation coefficients

	Missense	Synonymous	Intron	UTR
*r*_XN_ - *r*_NX_	-0.08	-0.27	-0.08	-0.12
*r*_XNN_ - *r*_NNX_	-0.19	-0.23	-0.14	-0.17
*r*_XNNN_ - *r*_NNNX_	-0.21_(<0.005)_	-0.14_(<0.05)_	-0.16_(<0.05)_	-0.16_(<0.05)_

See legend of Table 3 for further details

Interestingly, we found a directional 5’-3’ asymmetry: the linear correlation coefficients are always more negative when the flanking sequence is considered towards the 3’ end rather than towards the 5’ end. This effect is weak but statistically significant, with values in the -0.1 to -0.2 range, and seems to be independent of the gene region considered. Moreover, it extends beyond the dinucleotide level up to quadruplets at least.

This 5’-3’ asymmetry can be put in relation with the preferential direction of the charge transport along the DNA base stacking, which appears to be from 5’ to 3’ [27].

### Connection between vIP and pathogenicity of the SBSs

Electron hole transport can be expected to play an important role not only in the mutability of specific DNA sequences but also in the pathogenic effect of SBSs, since these substitutions can interfere, for example, with some repair mechanisms through the modification of the affinity for DNA-processing enzymes involved in DNA repair, or with basic cellular processes such as transcription and replication through the modification of targeted protein-DNA interactions.

The first quantity that we computed to gain insights into these matters is the linear correlation between the vIP of the motifs consisting of the wild-type bases and their flanking sequence and the difference in frequency (*Δν*) between pathogenic and neutral substitutions normalized by the frequency of the motif in the considered gene region. We grouped for this purpose pathogenic and likely pathogenic annotations, as well as benign and likely benign annotations.

As seen in Table 6, there is a small but significant correlation between vIP values and *Δν*, especially for transversions and missense mutations. In introns and for synonymous mutations, the correlations are not statistically significant, but this is probably due to the small number of annotated SBSs of these types, which prevents us to draw robust conclusions. In summary, pathogenic missense mutations are enriched with low vIP sequences, while we cannot conclude for other types of SBSs at this stage.

**Table 6:** Pearson’s linear correlation coefficients between the vIP of the wild type base surrounded by its flanking sequence and the difference in normalized frequency *Δν* between pathogenic and benign substitutions

	Doublets _XN_	Triplets _NXN_	Quadruplets _NXNN_
**Missense mutations in exons**			
All	-0.35	-0.18	-0.15_(<0.001)_
Transitions	-0.32	-0.10	-0.06_(<0.05)_
Transversions	-0.39	-0.35_(<0.0005))_	-0.27 $\phantom {\dot {i}\!}{~}_{(<10^{-6})}$
**Synonymous mutations in exons**			
All	-0.41	-0.20_(<0.05)_	-0.11
Transitions	-0.37	-0.16	-0.08
Transversions	-0.46	-0.21	-0.15
**Mutations in introns**			
All	0.20	0.17	0.06
Transitions	0.00	0.27	0.08
Transversions	0.10	-0.09	-0.02

See legend of Table 3 for further details

We also computed the correlation between the absolute values of the change in vIP between the wild type and mutated nucleotides (|*Δ*vIP|) and *Δν*. As we can see from Table 7, the correlation is positive and quite high: from 0.5 in UTRs up to 0.7 for intron mutations, and missense and synonymous mutations in exons. This suggests that a larger change in the vIP of the nucleotide base at which the mutation occurs is associated to a higher chance of the mutation to be pathogenic.

**Table 7:** Pearson’s linear correlation coefficients between the absolute values of the vIP changes upon SBSs (|*Δ*vIP|) and the difference in normalized frequency *Δν* between pathogenic and neutral substitutions

Missense	Synonymous	Intron	UTR
0.68_(<0.05)_	0.73_(<0.005)_	0.67_(<0.05)_	0.49

See legend of Table 3 for further details

However, when calculating the correlation between *Δν* and |*Δ*vIP| by taking into account the flanking sequences, no statistically significant result was found. This absence of correlation has several possible explanations. It could be due to the limited accuracy of the vIP calculations and the fact that differences in vIPs have a much larger relative error than vIPs themselves. This is moreover supported by the fact that, when using the calculated vIPs rather than the experimental ones, the correlations shown in Table 7 drop down to about 0.40. However, this result could also be taken to mean that the observed correlation between *Δν* and |*Δ*vIP| is not due to charge migration, but rather to other biophysical phenomena.

## Discussion

Our results show a clear overall anticorrelation between the vIP of short nucleotide sequences and the normalized frequencies of SBSs in these sequences. Note that the actual relation between vIPs and SBS frequencies appears to be non-linear (Fig. 1 and Additional file 1: Figure S1-S4), and we therefore also fitted the data using third-degree polynomials.

*On the average, the lower the vIP of a nucleobase sequence motif, the more frequent the SBSs in this motif and vice versa. This effect extends far beyond the dinucleotide units - it is still clearly visible for base sextuplets.*

These results support the fundamental role played by electron-hole transfer along the DNA stack in mutagenesis processes, which in some cases lead to deleterious phenotypes. The rationale behind this finding is that DNA charge transfer can interfere with different biomolecular mechanisms, triggering SBSs and/or preventing enzymatic-driven DNA repair mechanisms. Indeed, oxidative stress due to physical or chemical agents is likely to cause the extraction of an electron from DNA. Examples of such agents include exposure to ionizing radiation, long-wave ultraviolet light, and reactive oxygen species, just to mention some of them. The hole can then migrate and remain trapped in a minimum of the ionization potentials, and possibly trigger a base substitution. For example, guanine radical cations can react with water to form 8-oxo-guanine, which can trigger G:C →T:A transversions [28], while oxidative damage of the cytosine can undergo deamination and/or dehydration to a poorly repaired uracil, leading after replication to the G:C →A:T transition (for further information on oxidative damage of DNA bases, see [29]).

Furthermore, our analyses revealed a difference in tendency between Gua and Cyt substitutions on the one hand, and Ade and Thy SBSs on the other hand. For the former, a negative linear correlation between the vIP and the normalized frequency is observed, whereas for the latter the correlation coefficient is slightly positive or zero. Most interestingly, cytosine SBSs appear to be quite special: both their linear and non-linear correlations with vIPs are systematically better than for other bases, with linear and non-linear correlation coefficients of -0.7 and 0.9 for triplet SBSs. This is a very strong indication of the role that charge transport has in these type of substitutions.

Although the correlation coefficients between the vIP and the SBS frequency are very similar across the different gene regions, the slope of the linear regression lines varies. Indeed, the chance of having a base substitution in low-vIP sequences is much higher in introns than in exons and UTRs, and is also higher for synonymous than for missense substitutions. *A priori*, we did not expect differences in the mechanisms that trigger SBSs in the different gene regions, and certainly not between synonymous and missense substitutions. It can be argued that the latter result could be due to the lethality of some base substitutions that are thus not observed; such mutations are expected to be more often missense than synonymous, as the former modify the proteins and are thus more likely to be deleterious for their function. Or simply, the reason could be that there are more constraints on missense mutations due to their impact on the stability and function of the encoded proteins. Another possible explanation is that the synonymous codon selection is modulated by the constraint of maintaining specific electronic charge properties along the DNA.

Moreover, the larger slopes of the regression lines (in absolute value) observed for intron mutations compared to exon substitutions are related to the larger variance in their SBS frequencies (Table 1). This result can be put is relation with the observation that GGG triplets, which have the lowest vIP, are quite frequent near the 5’ termini of introns, where they have been suggested to serve as sacrificial anodes to protect the protein-coding part of the genes [30]. However, to definitely interpret our results, we need more insights into what occurs in the deep intronic regions, which mutagenesis mechanisms play a role, and how the correct functioning of the splicing process occurs [31].

Finally, we observed a difference between the linear correlation coefficients of SBSs occurring in 5’- and 3’-UTRs, as seen in Additional file 1: Table S3. 5’-UTR substitutions appear to be similar to synonymous mutations and 3’-UTR substitutions to missense mutations. The role of vIP is likely to be important in UTRs but has still to be fully understood. Such regions act to regulate and modulate the protein gene expression at the post-transcriptional level. They contain regulatory sequences that impact the mRNA stability, transport and translational efficiency. Charge transport in these regions can influence a variety of factors and its modification can thus lead to an impact on the normal functioning of the protein synthesis.

Another interesting result is the asymmetry with respect to the flanking sequence when computing the correlations between vIPs and SBS rates. Indeed, the correlation is always better when the mutated base is near the 5’ end of the considered sequence segment rather than near the 3’ terminus. This directional dependence could reflect the fact that the physical structure of the DNA stack is asymmetric. In particular, the overlap between successive bases differs between 5’-AB-3’ and 5’-BA-3’ configurations [32]. This leads in turn to nucleobase stack vIPs which are not symmetric with respect to sequence inversions, and thus to a DNA charge transport with a directional preference. Indeed, even though it remains difficult to test experimentally, electron hole transfer seems to be more efficient in the 5’-3’ than in the 3’-5’ direction [27]. Hence, if we assume that the charge transport mechanisms are strictly connected to mutagenesis processes, the vIP of the flanking sequence in the 3’ direction after the substituted nucleobase is expected to be better correlated with the observed SBS frequency. This corresponds indeed to what we observe.

Not only mutability appears to be related with electronic properties of DNA, but also pathogenicity. Indeed, we found a weak but statistically significant correlation between the pathogenicity of the SBSs and the vIP values of the wild-type bases and their flanking sequences. It is prevalently observed for missense mutations, but it has to be noted that the number of disease annotations for other types of variants is too low to yield reliable statistics. Note that this correlation is always larger for transversions than for transitions, and that the former are also more pathogenic in general.

Finally, we found quite a high correlation between the change in vIP between the wild-type and mutant nucleobases in absolute value (|*Δ* vIP |) and the pathogenicity of the SBSs. However, the correlation disappears when taking also into account the flanking sequences. This can be taken to mean that it is not the vIP that drives the correlation, but other characteristics of the four nucleobases. But it can also be due to the approximations done when calculating vIPs, which are described in the last Methods subsection. Indeed, differences in vIP values are tiny, and inaccuracies in vIPs have therefore a larger effect on *Δ*vIPs than on the vIPs themselves. More analyses are needed to settle this interesting issue.

Our results thus suggest that vIPs and the associated DNA charge transfer could be not only related to the mutation rate, but also to the pathogenicity of the mutations. This effect can be attributed to the modification of biophysical mechanisms such as DNA-protein binding, or base or nucleotide excision repair mechanisms [33, 34].

## Conclusion

Since the last two decades, it is becoming increasingly clear that DNA charge transport has a fundamental role in a wide range of biomolecular processes. Sometimes, cells use it for long-range biological sensing and signaling and it is then of vital importance, while in other cases oxidative stress induces electron holes that migrate along the base stack and can, for example, lead to single base mismatches via a wide series of chemical mechanisms, or interfere with the binding of specific proteins.

In this study we focused on the analysis of the SBS rates, and found a clear non-linear correlation with the vIP values of the wild-type bases and their neighboring sequence. This correlation differs for substitutions of Gua and Cyt bases and for Ade and Thy, and the slope of the regression line varies between exons and introns, and between synonymous and missense mutations. A 5’-3’ directional asymmetry of the correlation is another indication of the importance of DNA charge transfer in the mutagenesis mechanisms. We also found a weak relation between the difference in frequency of pathogenic and neutral mutations and the vIP values, observing that pathogenic mutations tend to be embedded in base sequences of lower vIP. A detailed understanding of these results is currently out of reach, but we guess that it would constitute an important step toward the understanding of DNA mutability and how mutations cause pathogenic phenotypes.

Several points remain to be clarified and further explored to get a clearer picture. Our first plan is to extend our analysis to longer base sequences, in view of understanding up to which point there is a signal between SBS frequencies and vIP values. Indeed, even though a electron hole trapped in a potential well remains localized in a region composed of 2-4 nucleobases, it can migrate much further away, over distances up to 200 Å [16]. The calculation of the vIP of longer nucleobase stacks is increasingly computationally demanding, but cannot be avoided. As a test, we estimated the vIP of base sequences in two ways, either by calculating directly the vIP of the whole segment using MP2, or by estimating it as the average of the vIPs of the single nucleobases that compose it. We found that SBS frequencies correlate better with the vIP values obtained in the first manner, as shown in Additional file 2: Table S4. The stacked base structure has thus definitely to be considered if we want to explore the behavior of the charge transfer at the quantum level.

vIP values are known to change as a function of the DNA structure [22], so that trapped holes can start migrating again upon (even small) DNA conformational modifications. In this study, we considered only DNA segments in standard B-conformations, but our analysis can easily be extended to A-conformations and other conformations observed in experimental structures. It would also be worthwhile to take into account different nucleotide modifications, such as the 5-methylation of cytosines, as they obviously impact on the vIP values and thus on the derivation of our results.

Another perspective consists in focusing on SBSs occurring in some specific diseases such as cancer and to analyze the link between the observed mutational signatures and vIP values. In particular, the extension of our investigation to datasets such as COSMIC [35], in which somatic cancer mutations are collected, could certainly help to get more insights into the role of the vIP in the mutation processes. It will be interesting to connect our findings to the 30 mutational signatures that have been observed [35] and are unique combinations of mutations occurring across the spectrum of human cancer types. Moreover, since such variants are not subject to selective pressure, which can be suspected to influence the correlations between the vIP values and the frequency of the DNA base motifs, we could expect even stronger signals compared to those observed in the present analysis.

All the analyses proposed here aim to better understand the mutagenesis processes and how oxidative stress caused by chemical and physical agents affect the electron-hole transfer in DNA and lead to nucleobase substitutions and pathogenic phenotypes.

## Methods

### Dataset of mutations

The SBS data were extracted from the Single Nucleotide Polymorphism Database (dbSNP) [36]. We focused on variants in gene regions, and for all of them, we collected the wild type and mutant nucleotides, the thirty flanking nucleotides, *i.e.* fifteen in the 5’ direction and fifteen in the 3’ direction, the region of the gene in which the mutation is inserted (exon, intron, 5’-UTR, or 3’-UTR). If the variant is in an exon region, we moreover identified the exact position of the mutated site with respect to the translated codon in order to determine whether it corresponds to a missense or a synonymous mutation.

Our final SBS dataset is defined as the subset of these SBSs for which the clinical phenotype (benign, likely benign, likely pathogenic, pathogenic, variant of unclear significance,...) is annotated in the ClinVar database [37].

Three quarters of the 190,000 variants from our SBS dataset occur in exon regions, while the remaining are variants in introns (about 25,000) and in UTR regions (30,000 in 3’-UTR and 8,000 in 5’-UTR). Among the exon SBSs, 72% are missense variants and 28% synonymous. Only one third of the variants have an established phenotypic effect (benign, likely benign, likely pathogenic, pathogenic), while the remaining two thirds of the variants have uncertain significance or other annotations.

### Frequency of nucleobase motifs

We computed the normalized frequency of base pairs, doublets, triplets, up to sextuplets by dividing the frequency of their occurrence in the SBS dataset by their frequency of observation in each specific gene region (exon, intron, UTR). To estimate the latter frequencies, we used the UCSC Genome browser [38]. We first retrieved the positional table of the selected regions in the genome assembly GRCh38.p12, and then dowloaded the corresponding sequences from which we computed all the motif frequencies.

### Vertical ionization potentials of nucleobase sequences

For computing the vIP of nucleobase sequences, we used the procedure set up in [22]. In a first stage, the four nucleobases Ade, Cyt, Gua, and Thy were considered separately. Their sugar cycle and phosphate group were omitted, and the glycosidic bond was replaced by a hydrogen atom. Their initial geometries were taken from the molecular-modeling program package Insight 2000 (Accelrys Inc.). These geometries were then optimized at second-order Møller-Plesset perturbation theory (MP2) [39] using the split-valence basis set 6-31G* with added d polarization functions on non-hydrogen atoms [40]. The Gaussian 09 program suite [41] was used for these, and all subsequent, quantum chemistry calculations.

The energy in gas phase of these optimized geometries was computed for the neutral species and for the radical cationic species, with one missing electron. The calculations for cationic molecules were performed with restricted open-shell procedures to prevent spin contamination problems. The vIP was defined as the difference between the energy of the cationic and neutral species.

In a second stage, we considered all possible single-stranded nucleobase stacks in standard B-conformation containing two, three or four nucleobases. The geometries of the stacks were taken from early fiber X-ray diffraction studies [42], using Insight 2000 (Accelrys Inc.). The isolated nucleobases, individually optimized at the MP2/6-31G* level, were superimposed onto the original bases forming the stacks, so as to minimize their root mean square deviation of atomic positions using the U3BEST algorithm [43]. The gas phase energies of these stacks of optimized nucleobases, for the neutral and radical cationic species, were calculated at MP2/6-31G* level, using the same geometry for both species. The vIP was computed as the difference between the energy of the radical cationic and neutral species, both adopting the same geometry.

Note that we used MP2 to calculate the vIPs rather than the hybrid density functional theory method M06-2X [44] that we used in [22]. Indeed, although the vIPs of the single nucleobases are closer to the experimental values when using M06-2X, the results on nucleobase stacks appear to be better when using MP2, as already described before [45]. For example, the GGG triplets have, as expected, the lowest vIP of all triplets with MP2, but not with M06-2X.

### Approximations in the vIP calculations

We made several approximations when computing the vIPs of the nucleobase stacks. First, we omitted the sugar-phosphate backbone. Of course, their inclusion would require too much computer time and memory, but there are other justifications. Calculations on Cyt and Thy indicated that the vIP values change according to the presence or absence of the sugar and phosphate moieties in gas phase, but much less in an aqueous solvent due to screening effects [46]. Moreover, *ab initio* calculations and experimental data showed that the lowest ionization pathway comes from the nucleobase stacks and not from the sugar-phosphate backbone [46, 47].

The second approximation we made is to perform all calculations in gas phase. We made this choice to reduce computer time, but also because such calculations seem to be suitable for comparing the vIP values of various nucleobase stack sequences. Indeed, calculations on individual nucleobases highlighted the effect of the solvent in lowering the vIP values while maintaining the relative ordering between the bases [48, 49]. Moreover, dropping both the sugar-phosphate backbone and the solvent have opposite effects that tend to cancel out [46].

We also calculated vIPs of single-stranded rather than double-stranded nucleobase stacks. This is justified by the fact that electron holes migrate along a single strand and jump only rarely to the complementary strand [50]. Moreover, we assumed a fixed B-conformation, although DNA molecules have some flexibility and the vIPs of nucleobase stacks in A- and B-conformations have been shown to differ significantly [22].

### Correlation coefficients and statistical tests

When we refer to the linear correlation coefficient of two variables *X* and *Y*, we mean the Pearson correlation coefficient. We also defined a non-linear correlation coefficient, obtained using a polynomial function of third degree:

1 $$ f(X) = a + b X^{-1} + c X^{-2} + d X^{-3}  $$

where the parameters *a*, *b*, *c*, and *d* are identified so as to minimize the root mean square deviation between *f*(*X*) and *Y*. The non-linear correlation coefficient is the Pearson correlation coefficient computed between *f*(*X*) and *Y*.

To check the statistical significance of correlations or pairs of correlations, we performed a hypothesis test on the bivariate sample against the null hypothesis. This was done by applying the Fischer z-transformation to the sample, followed by a z-test.

## Additional files

Additional file 1 **Table S1**. Non-linear correlation coefficients between the vIP of the wild-type nucleotide motifs and the normalized SBS frequency in the different gene regions. **Table S2**. Statistical power and confidence interval for the linear correlation coefficients between the vIPs of the wild-type nucleotide motifs and the normalized SBS frequencies. **Table S3**. Pearson’s linear correlation coefficients between the vIP of the wild-type nucleotides and their flanking base sequences and the normalized SBS frequency in UTR-5 and UTR-3 regions. **Figure S1-S4.** Normalized frequency of observation of missense, synonymous, intron and UTR SBSs as a function of the vIP (in eV) of nucleobase quintuplets NNXNN, where X indicates the SBS and N any base. (PDF 985 kb)

Additional file 2 **Table S4**. Excel file with the normalized and the standard SBS frequencies for single bases (sheet-Single), base doublets (sheet-Doublet), triplets (sheet-Triplet), quadruplets (sheet-Quadruplet), and quintuplets (sheet-Quintuplet). In (sheet-ObsExp), the frequency of observation of the motifs in the different gene regions are reported. (sheet-AverageVSComp) contains the comparison between the vIPs of the quadruplets computed using MP2 with those computed by averaging the MP2 vIPs of the overlapping triplets or doublets, and of the constituent nucleobases. Finally, in (sheet-MP2vsM06-2X), the comparison of the triplet vIPs computed with MP2 with those computed with M06-2X is reported. (XLSX 330 kb)

## Data Availability

The full contents of the supplementary information are available online at https://bmcgenomics.biomedcentral.com/

## Abbreviations

mC: Methylated cytosine
MP2: Møller-Plesset perturbation theory
SBSs: Single base substitutions
UTR: Untranslated region
vIP: Vertical ionization potential
