# Supplementary material for: Relation between DNA ionization potentials, single base substitutions and pathogenic variants
Source: BMC Genomics. 2019 Jul 16;20(Suppl 8):551. doi: 10.1186/s12864-019-5867-y (PMC6631442; doi:10.1186/s12864-019-5867-y)
Supplement: Supplementary file 1 — Table S1. Non-linear correlation coefficients between the vIP of the wild-type nucleotide motifs and the normalized SBS frequency in the different gene regions. Table S2. Statistical power and confidence interval for the linear correlation coefficients between the vIPs of the wild-type nucleotide motifs and the normalized SBS frequencies. Table S3. Pearson’s linear correlation coefficients between the vIP of the wild-type nucleotides and their flanking base sequences and the normalized SBS frequency in UTR-5 and UTR-3 regions. Figure S1-S4. Normalized frequency of observation of missense, synonymous, intron and UTR SBSs as a function of the vIP (in eV) of nucleobase quintuplets NNXNN, where X indicates the SBS and N any base. (PDF 985 kb) [file 12864_2019_5867_MOESM1_ESM.pdf]

Supplementary Information

F. Pucci, M. Rooman

Relation between DNA ionization potentials, single base substitutions and pathogenic variants.

- **Table S1**. Non-linear correlation coefficients between the vIP of the wild-type nucleotide motifs and the normalized SBS frequency in the different gene regions.
- **Table S2**. Statistical power and confidence interval for the linear correlation coefficients between the vIPs of the wild-type nucleotide motifs and the normalized SBS frequencies.
- **Table S3**. Pearson's linear correlation coefficients between the vIP of the wild-type nucleotides and their flanking base sequences and the normalized SBS frequency in UTR-5 and UTR-3 regions.
- **Table S4**. Excel file with the normalized and the standard SBS frequencies for single bases (sheet-Single), base doublets (sheet-Doublet), triplets (sheet-Triplet), quadruplets (sheet-Quadruplet), and quintuplets (sheet-Quintuplet). In (sheet-ObsExp), the frequency of observation of the motifs in the different gene regions are reported. (sheet-AverageVSComp) contains the comparison between the vIPs of the quadruplets computed using MP2 with those computed by averaging the MP2 vIPs of the overlapping triplets or doublets, and of the constituent nucleobases. Finally, in (sheet-MP2vsM06-2X), the comparison of the triplet vIPs computed with MP2 with those computed with M06-2X is reported.
- **Figures S1-S4** Normalized frequency of observation of missense, synonymous, intron and UTR SBSs as a function of the vIP (in eV) of nucleobase quintuplets NNXNN, where X indicates the SBS and N any base. The four wild-type bases, X=Gua, Ade, Cyt and Thy, are considered separately.

Table S1. Non-linear correlation coefficients between the vIP of the wild-type nucleotide motifs and the normalized SBS frequency in the different gene regions. X indicates the position of the mutated nucleobase and N any base. The correlation coefficients that are statistically significant and for which the null hypothesis is rejected are underlined, with the P-values below $\alpha = 0.05$ reported in parentheses. Moreover, the ANOVA F-ratio has been computed for each entry and the associated P-values are always < 0.005 (not shown). The correlations are thus highly statistically significant.

WT	$\text{Triplet}_{\text{NXN}}$	$\text{Quadruplet}_{\text{NXNN}}$	$\text{Quintuplet}_{\text{NNXNN}}$	$\text{Sextuplet}_{\text{NNXNNN}}$
		**Missense mutations in exons**		
All	$\underline{0.61}_{(<0.001)}$	$\underline{0.53}_{(<0.01)}$	$\underline{0.47}_{(<10^{-12})}$	$\underline{0.42}_{(<10^{-46})}$
G	$\underline{0.63}_{(<0.01)}$	$\underline{0.44}_{(<0.001)}$	$\underline{0.35}_{(<10^{-7})}$	$\underline{0.25}_{(<10^{-13})}$
C	$\underline{0.93}_{(<0.05)}$	$\underline{0.83}_{(<10^{-5})}$	$\underline{0.75}_{(<10^{-15})}$	$\underline{0.62}_{(<10^{-52})}$
A	$\underline{0.65}_{(<0.05)}$	$\underline{0.43}_{(<0.0001)}$	$\underline{0.36}_{(<10^{-9})}$	$\underline{0.26}_{(<10^{-26})}$
T	0.19	$\underline{0.30}_{(<0.05)}$	$\underline{0.17}_{(<0.05)}$	$\underline{0.13}_{(<0.01)}$
		**Synonymous mutations in exons**		
All	$\underline{0.56}_{(<0.05)}$	$\underline{0.56}_{(<10^{-7})}$	$\underline{0.49}_{(<10^{-24})}$	$\underline{0.43}_{(<10^{-109})}$
G	$\underline{0.76}_{(<0.001)}$	$\underline{0.55}_{(<0.00005)}$	$\underline{0.47}_{(<10^{-13})}$	$\underline{0.34}_{(<10^{-31})}$
C	$\underline{0.93}_{(<0.005)}$	$\underline{0.73}_{(<0.005)}$	$\underline{0.70}_{(<10^{-11})}$	$\underline{0.60}_{(<10^{-50})}$
A	0.11	$\underline{0.37}_{(<0.01)}$	0.07	$\underline{0.21}_{(<10^{-8})}$
T	0.28	0.22	$\underline{0.27}_{(<0.0005)}$	$\underline{0.16}_{(<0.00005)}$
		**Mutations in introns**		
All	$\underline{0.39}_{(<0.001)}$	$\underline{0.55}_{(<10^{-7})}$	$\underline{0.47}_{(<10^{-27})}$	$\underline{0.43}_{(<10^{-89})}$
G	$\underline{0.74}_{(<0.005)}$	$\underline{0.41}_{(<0.05)}$	$\underline{0.36}_{(<10^{-7})}$	$\underline{0.23}_{(<10^{-10})}$
C	$\underline{0.63}_{(<0.01)}$	$\underline{0.85}_{(<10^{-10})}$	$\underline{0.74}_{(<10^{-27})}$	$\underline{0.70}_{(<10^{-97})}$
A	0.67	$\underline{0.56}_{(<10^{-6})}$	$\underline{0.26}_{(<0.00005)}$	$\underline{0.13}_{(<10^{-8})}$
T	0.74	$\underline{0.26}_{(<0.05)}$	$\underline{0.15}_{(<0.05)}$	$\underline{0.17}_{(<0.001)}$
		**Mutations in UTRs**		
All	$\underline{0.50}_{(<0.05)}$	$\underline{0.50}_{(<0.001)}$	$\underline{0.44}_{(<10^{-14})}$	$\underline{0.41}_{(<10^{-52})}$
G	$\underline{0.61}_{(<0.001)}$	$\underline{0.40}_{(<0.05)}$	$\underline{0.31}_{(<0.0001)}$	$\underline{0.16}_{(<0.0001)}$
C	$\underline{0.91}_{(<0.05)}$	$\underline{0.82}_{(<10^{-7})}$	$\underline{0.77}_{(<10^{-23})}$	$\underline{0.68}_{(<10^{-72})}$
A	$\underline{0.75}_{(<0.005)}$	$\underline{0.61}_{(<0.0005)}$	$\underline{0.50}_{(<10^{-11})}$	$\underline{0.31}_{(<10^{-20})}$
T	0.41	$\underline{0.31}_{(<0.05)}$	$\underline{0.28}_{(<10^{-5})}$	$\underline{0.13}_{(<0.0005)}$

Table S2. Statistical power and confidence interval for the linear correlation coefficients between the vIPs of the wild-type nucleotide motifs and the normalized SBS frequencies. The correlation coefficients that are statistically significant and for which the null hypothesis is rejected are underlined, with the P-values below $\alpha = 0.05$ reported in parentheses. X indicates the position of the mutated nucleobase and N any base.

Motif	Correlation coefficient	Statistical power	Confidence interval
**Missense mutations in exons**			
$\text{Doublet}_{\text{XN}}$	-0.39	0.351	[0.13,-0.74]
$\text{Triplet}_{\text{NXN}}$	$\underline{-0.39}_{(<0.05)}$	0.915	[-0.16,-0.58]
$\text{Quadruplet}_{\text{NXNN}}$	$\underline{-0.35}_{(<0.01)}$	1.000	[-0.24,-0.45]
$\text{Quintuplet}_{\text{NNXNN}}$	$\underline{-0.34}_{(<10^{-13})}$	1.000	[-0.29,-0.39]
**Synonymous mutations in exons**			
$\text{Doublet}_{\text{XN}}$	-0.48	0.531	[0.02,-0.79]
$\text{Triplet}_{\text{NXN}}$	$\underline{-0.42}_{(<0.005)}$	0.954	[-0.20,-0.60]
$\text{Quadruplet}_{\text{NXNN}}$	$\underline{-0.39}_{(<10^{-8})}$	1.000	[-0.28,-0.49]
$\text{Quintuplet}_{\text{NNXNN}}$	$\underline{-0.38}_{(<10^{-29})}$	1.000	[-0.33,-0.43]
**Mutations in introns**			
$\text{Doublet}_{\text{NXN}}$	-0.39	0.426	[0.13,-0.74]
$\text{Triplet}_{\text{NXN}}$	$\underline{-0.42}_{(<0.005)}$	0.954	[-0.20,0.60]
$\text{Quadruplet}_{\text{NXNN}}$	$\underline{-0.38}_{(<10^{-5})}$	1.000	[-0.27,-0.48]
$\text{Quintuplet}_{\text{NNXNN}}$	$\underline{-0.36}_{(<10^{-24})}$	1.000	[-0.31,-0.41]
**Mutations in UTRs**			
$\text{Doublet}_{\text{XN}}$	-0.43	0.488	[0.08,-0.76]
$\text{Triplet}_{\text{NXN}}$	$\underline{-0.39}_{(<0.05)}$	0.915	[-0.16,-0.58]
$\text{Quadruplet}_{\text{NXNN}}$	$\underline{-0.35}_{(<0.005)}$	1.000	[-0.24,-0.45]
$\text{Quintuplet}_{\text{NNXNN}}$	$\underline{-0.34}_{(<10^{-16})}$	1.000	[-0.29,-0.39]

Table S3. Pearson's linear correlation coefficients between the vIP of the wild-type nucleotides and their flanking base sequences and the normalized SBS frequency in UTR-5 and UTR-3 regions. The correlation coefficients that are statistically significant and for which the null hypothesis is rejected are underlined, with the P-values below $\alpha = 0.05$ reported in parentheses. X indicates the position of the mutated nucleobase and N any base.

WT	$\text{Doublet}_{\text{XN}}$	$\text{Triplet}_{\text{NXN}}$	$\text{Quadruplet}_{\text{NXNN}}$	$\text{Quintuplet}_{\text{NNXNN}}$
**Mutations in 5'-UTRs**				
All	$\underline{-0.52}_{(<0.005)}$	$\underline{-0.47}_{(<10^{-6})}$	$\underline{-0.44}_{(<10^{-14})}$	$\underline{-0.41}_{(<10^{-50})}$
G	-0.70	$\underline{-0.62}_{(<0.01)}$	$\underline{-0.39}_{(<0.005)}$	$\underline{-0.35}_{(<10^{-7})}$
C	-0.85	$\underline{-0.73}_{(<0.001)}$	$\underline{-0.66}_{(<10^{-12})}$	$\underline{-0.62}_{(<10^{-40})}$
A	0.22	0.11	-0.03	0.00
T	-0.96	-0.41	$\underline{-0.42}_{(<0.0005)}$	$\underline{-0.32}_{(<10^{-9})}$
**Mutations in 3'-UTRs**				
All	-0.39	$\underline{-0.38}_{(<0.05)}$	$\underline{-0.33}_{(<0.05)}$	$\underline{-0.31}_{(<10^{-7})}$
G	0.06	-0.45	$\underline{-0.30}_{(<0.05)}$	$\underline{-0.22}_{(<0.05)}$
C	-0.76	$\underline{-0.70}_{(<0.05)}$	$\underline{-0.56}_{(<0.05)}$	$\underline{-0.53}_{(<10^{-8})}$
A	0.55	$\underline{0.63}_{(<0.01)}$	$\underline{0.54}_{(<0.0005)}$	$\underline{0.50}_{(<10^{-14})}$
T	-0.05	0.11	0.07	$\underline{0.09}_{(<0.05)}$

Missense mutations in exons

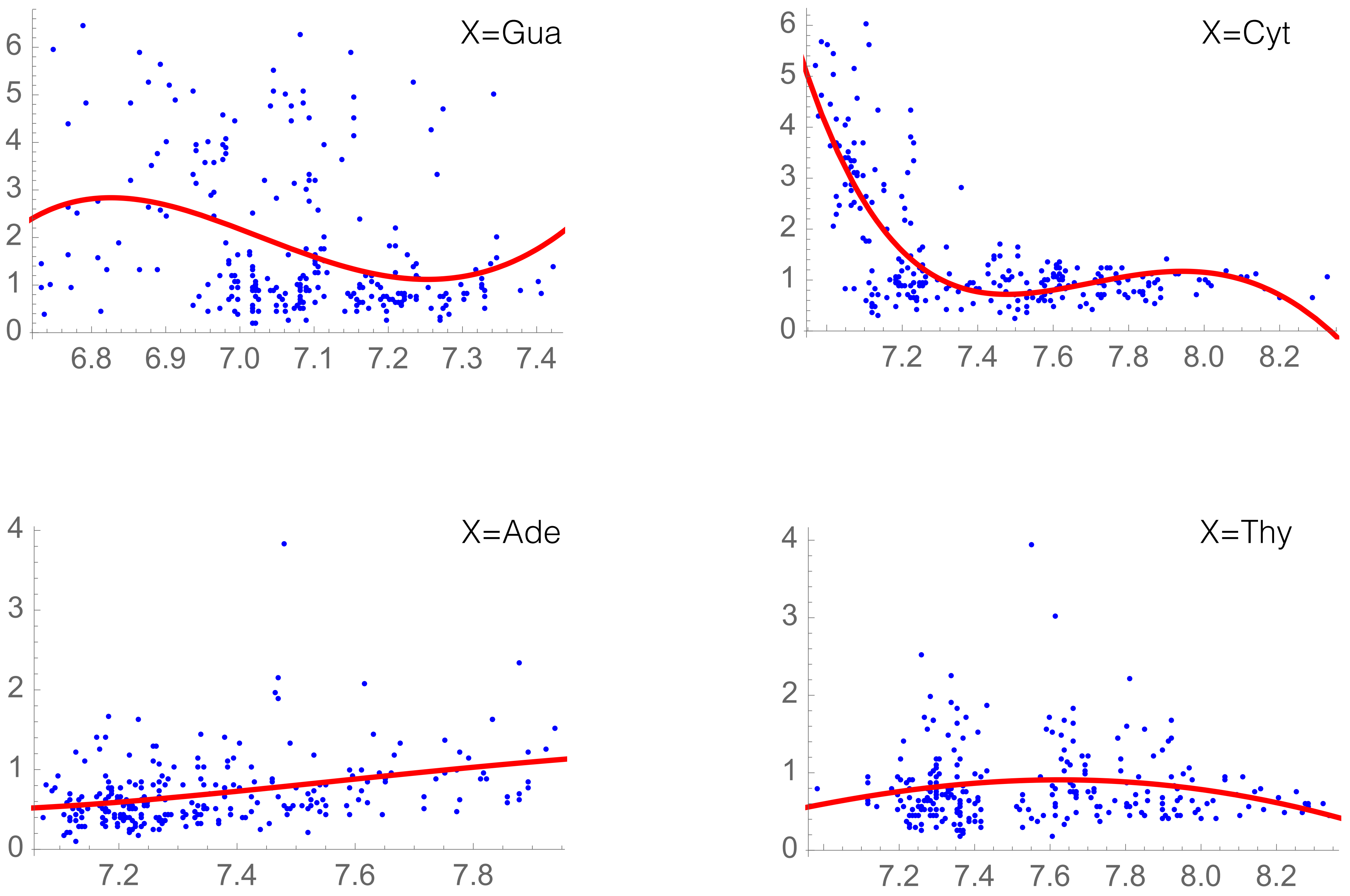

Figure S1. Normalized frequency of observation of missense SBSs as a function of the vIP (in eV) of nucleobase quintuplets NNXNN, where X indicates the SBS and N any base. The four wild-type bases, X=Gua, Ade, Cyt and Thy, are considered separately.

Synonymous mutations in exons

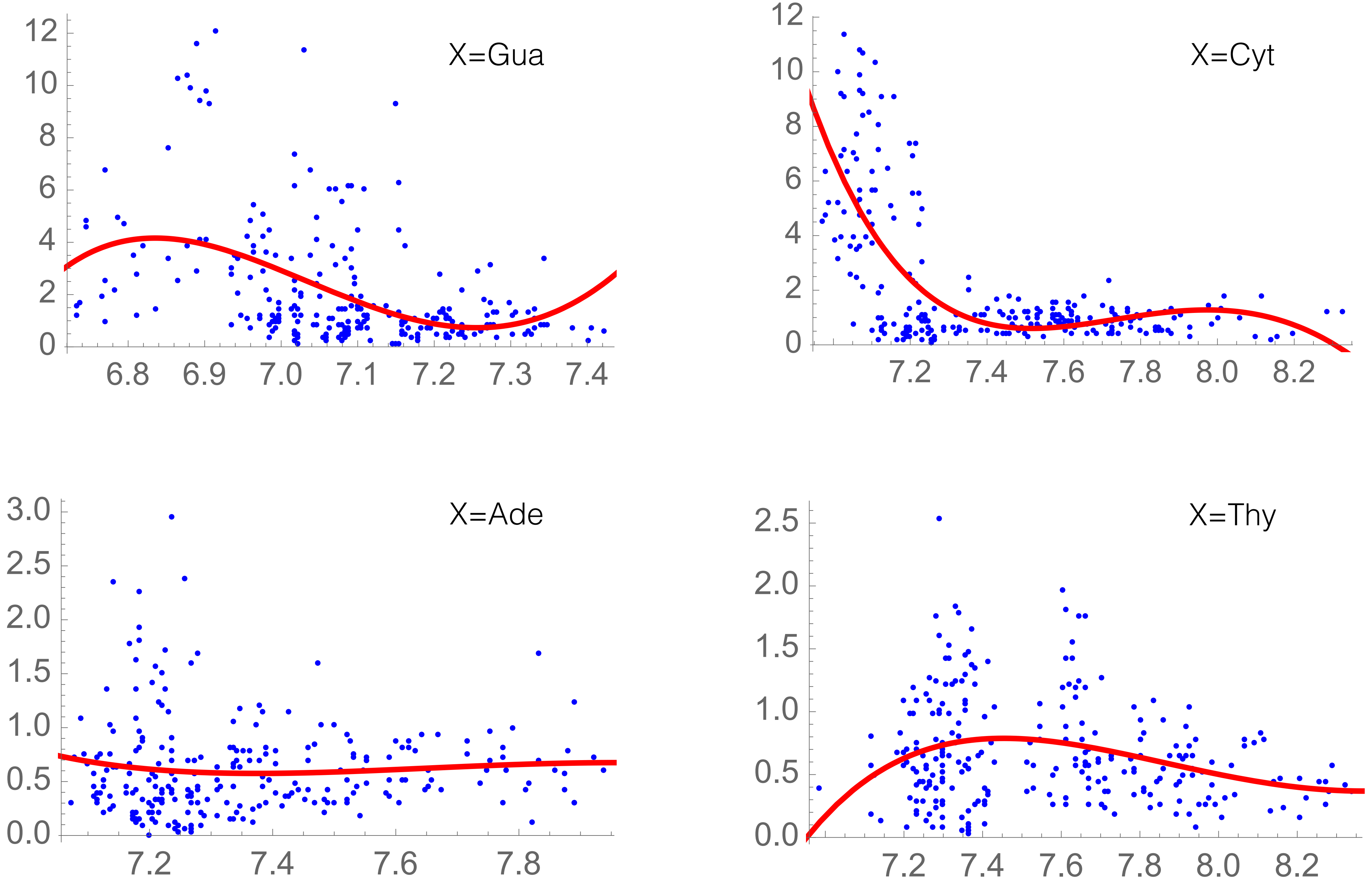

Figure S2. Normalized frequency of observation of synonymous SBSs as a function of the vIP (in eV) of nucleobase quintuplets NNXNN, where X indicates the SBS and N any base. The four wild-type bases, X=Gua, Ade, Cyt and Thy, are considered separately.

Mutations in introns

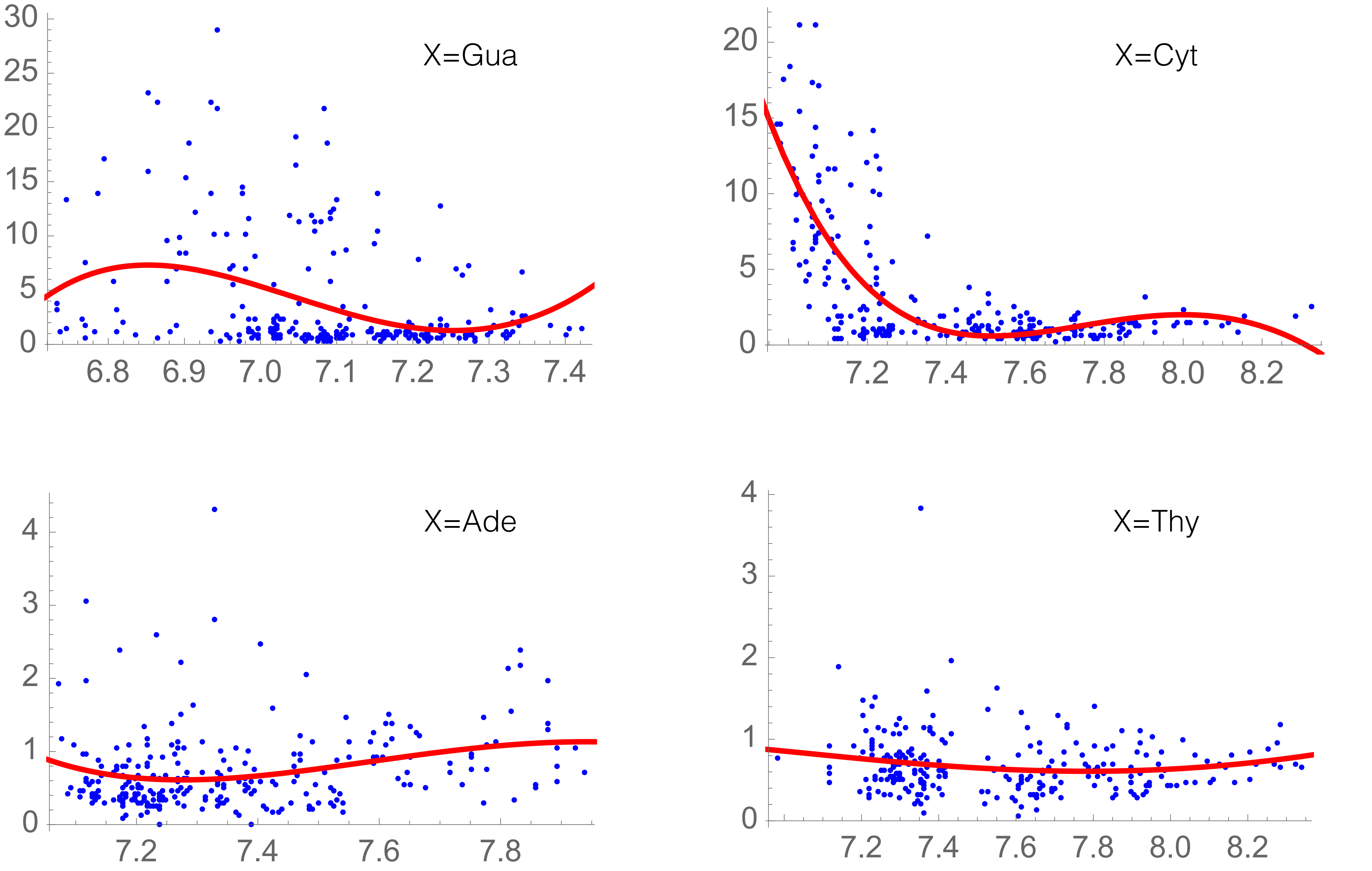

Figure S3.Normalized frequency of observation of intron SBSs as a function of the vIP (in eV) of nucleobase quintuplets NNXNN, where X indicates the SBS and N any base. The four wild-type bases, X=Gua, Ade, Cyt and Thy, are considered separately.

Mutations in UTRs

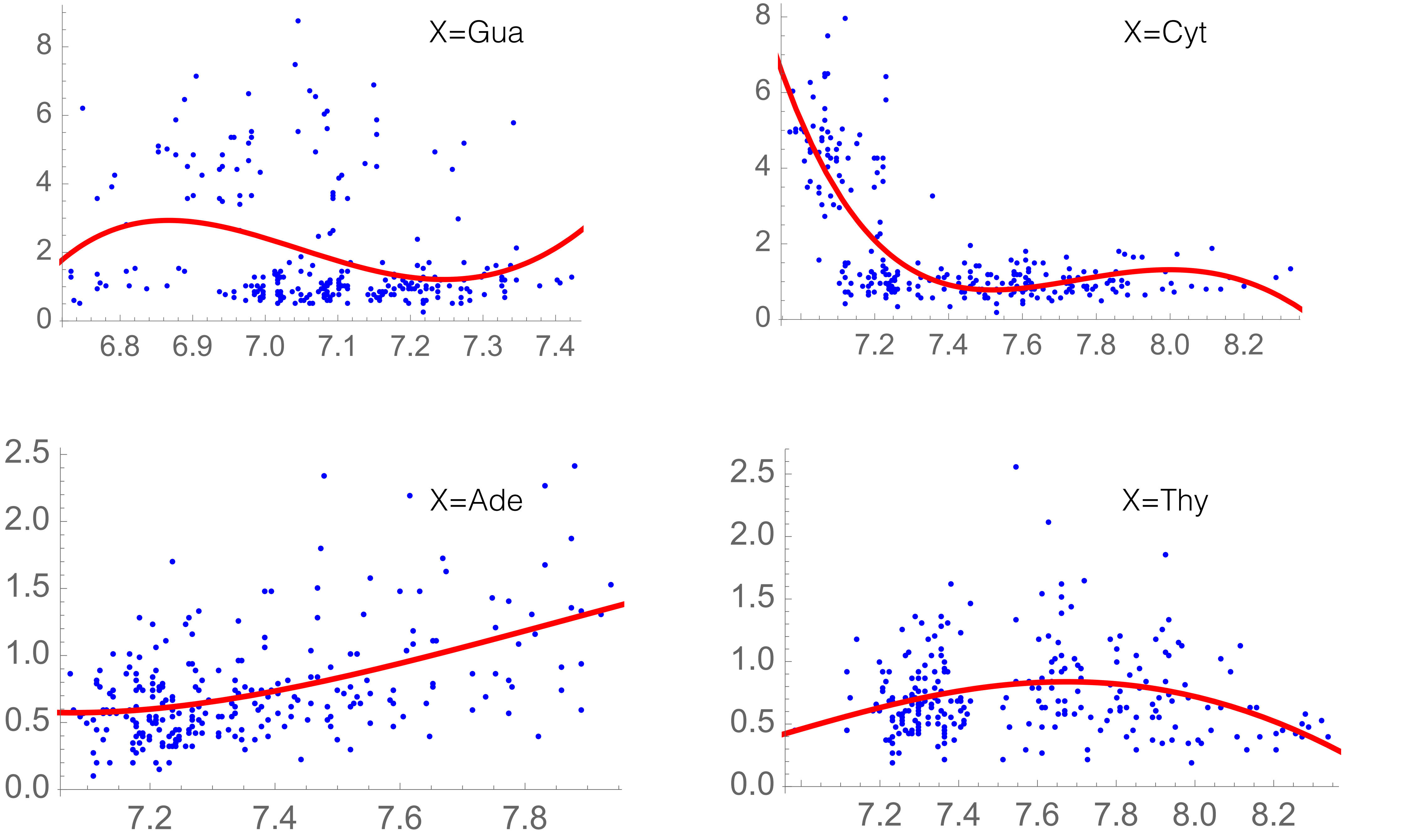

Figure S4. Normalized frequency of observation of UTR SBSs as a function of the vIP (in eV) of nucleobase quintuplets NNXNN, where X indicates the SBS and N any base. The four wild-type bases, X=Gua, Ade, Cyt and Thy, are considered separately.
